# A Metacontrol Perspective on Neurocognitive Atypicality: From Unipolar to Bipolar Accounts

**DOI:** 10.3389/fpsyt.2022.846607

**Published:** 2022-06-23

**Authors:** Lorenza S. Colzato, Christian Beste, Wenxin Zhang, Bernhard Hommel

**Affiliations:** ^1^Cognitive Neurophysiology, Department of Child and Adolescent Psychiatry, Faculty of Medicine, TU Dresden, Dresden, Germany; ^2^Cognitive Psychology, Faculty of Psychology, Shandong Normal University, Jinan, China

**Keywords:** ASD, autism, tics, ADHD, metacontrol

## Abstract

Standard clinical and psychiatric thinking follows a unipolar logic that is centered at “normal” conditions characterized by optimal performance in everyday life, with more atypical conditions being defined by the (degree of) absence of “normality.” A similar logic has been used to describe cognitive control, assuming that optimal control abilities are characterized by a strong focus on the current goal and ignorance of goal-irrelevant information (the concept of willpower), while difficulties in focusing and ignoring are considered indications of the absence of control abilities. However, there is increasing evidence that willpower represents only one side of the control coin. While a strong focus on the current goal can be beneficial under some conditions, other conditions would benefit from a more open mind, from flexibility to consider alternative goals and information related to them. According to the metacontrol model, people can vary in their cognitive processing style, on a dimension with the extreme poles of “persistence” on the one hand and “flexibility” on the other. Whereas a high degree of persistence corresponds to the original idea of cognitive control as willpower, with a strong focus on one goal and the information related to it, a high degree of flexibility is characterized by a more integrative, less selective and exclusive processing style, which facilitates switching between tasks, ideas, and actions, and taking into consideration a broader range of possibilities. We argue that this approach calls for a more bipolar account in the clinical sciences as well. Rather than considering individuals as typical or atypical, it would theoretically and practically make more sense to characterize their cognitive abilities in terms of underlying dimensions, such as the persistence/flexibility dimension. This would reveal that possible weaknesses with respect to one pole, such as persistence, and tasks relying thereupon, may come with corresponding strengths with respect to the other pole, such as flexibility, and respective tasks. We bolster our claim by discussing available evidence suggesting that neurodevelopmental atypicality often comes with weaknesses in tasks related to one pole but strengths in tasks related to the other.

## The Unipolar View of Standard Clinical and Psychiatric Thinking

Clinicians and psychiatrists usually follow what can be characterized as a unipolar logic, which focuses on “typical” or “normal” conditions that refer to average or optimal performance and behavior in everyday life, while other (“atypical” or “deviant”) conditions are defined by the absence of behavioral or performance-related “normality.” According to this unipolar logic, those whose mental performance or overt behavior digress from what is considered average humans are considered “abnormal” or “deviant” and thus in need of treatment—with the goal of reducing the gap between their behavior and that of the average population. This view is consolidated in our society notwithstanding the fact that the degree of deviation from average mental performance is often not clear and justified objectively, but rather reflects present cultural and societal norms and expectations (1, 2). Several other disciplines share this unipolar logic as well, including linguistics, pedagogy, and the social sciences, which commonly characterize atypical individuals in terms of the difficulties and challenges they face in trying to comply with societal norms and criteria, and to meet societal expectations, in terms of the deficiencies they have, and the limitations they possess (3–6). The downside of this unipolar logic is that atypical behaviors become stigmatized, with potentially serious personal and social consequences for the people manifesting them (4, 7–10).

In contrast to the impression one gets from the clinical and psychiatric descriptions of individuals meeting psychiatric diagnostic criteria, there is increasing evidence that these individuals may also have specific mental and physical strengths, suggesting that atypicality can go both ways. Especially in the field of autism spectrum disorder (ASD), attention deficit and hyperactivity disorders (ADHD), and tic disorders, new approaches are suggesting that some aspects of these conditions can be adaptive, or even hyper-adaptive, rather than sub- average or dysfunctional [e.g., (4, 11–13)]. This raises the possibility that psychiatric conditions are insufficiently captured by a strong focus on negative deviance (i.e., poorer performance than average on particular dimensions) but should be complemented by a focus on possible positive deviance (i.e., better performance than average on other dimensions). In this hypothesis and theory article, we argue that this is indeed the case, which in our view requires a structural modification of our view on atypicality and deviance, and the carriers of atypical and deviant behavior. In particular, we claim that, in order to get a more balanced view on psychiatric conditions, the unipolar perspective that distinguishes between typical and atypical, between normal and deviant, needs to be replaced by a systematic theoretical framework that emphasizes bipolar dimensions and characterizations of human mental performance.

We will develop our argument as follows. First, we will discuss recent theoretical developments in our understanding of human cognitive control, a neurocognitive function that is closely associated with, and implicated by various neurocognitive atypicalities. As we will try to show, theorizing on cognitive control shares the unipolar perspective with clinical/psychiatric theorizing, by defining optimal conditions on the one hand and various degrees of deviance from them on the other. Interestingly, however, there is increasing evidence that this perspective is too limited and so it has given way to a more bipolar perspective on cognitive control, as expressed by the metacontrol model of Hommel (14) and Hommel and Colzato (15). Second, we will then use this development as a template that we apply to clinical/psychiatric theorizing on neurocognitive atypicality, and argue that this theorizing would also benefit from a more bipolar view. Third, we present preliminary but encouraging evidence from neurodevelopmental conditions that a bipolar perspective on atypicality might indeed be feasible and inspiring.

## From Unipolar to Bipolar Accounts of Cognitive Control: The Metacontrol Model

In order to motivate our plea for a move from unipolar to bipolar logic with respect to psychiatric conditions, we would like to describe a similar move in a research field that is of considerable relevance for many psychiatric conditions, including ASD, ADHD, obsessive-com pulsive disorder (OCD), and various kinds of tics: the investigation of cognitive control and executive functions. Our historical view of cognitive control (or, in historical terms: of the human will) exhibits an equally strong reliance on unipolar logic as our theoretical thinking about psychiatric deviance. In particular, optimal cognitive-control abilities are assumed to be characterized by a strong focus on the current goal and the ideally complete ignorance of goal-irrelevant information (the concept of willpower). Accordingly, any difficulties in focusing on what is currently important and ignoring of what is not are considered indications of suboptimal, weak control abilities (16–18) which, depending on the degree of weakness, are responsible for failures of self-control and resulting problematic atypical behaviors (18, 19). Hence, the success (vs. failure) of the exclusive focusing on what is currently relevant, and the corresponding ignorance of what is currently not, is assumed to characterize people’s individual cognitive-control abilities and the degree of deviant behavior they might show. In other words, cognitive control is assumed to vary from strong to weak, implying some cutoff point at which sufficiently suboptimal, deviant behavior becomes likely.

In the last decade, however, this unipolar view has been challenged by conceptual considerations and empirical evidence. As it turns out, willpower represents only one side of the control coin: While a strong focus on the current goal can be beneficial under some conditions, other conditions would benefit from a more open mind, from a person’s flexibility to consider alternative goals and information related to them. In fact, a species that would embody the ideal of traditional thinking about cognitive control would be bound to become extinct within very short time: as much as the hunter needs to keep her prey in mind in order to follow it through thick and thin until the hunt was successful, it would be unwise to ignore the task-irrelevant predator on the way or to continue the hunt if it’s success becomes unlikely. Hence, prioritization of goals seems more adaptive than exclusive focus on one, and not acting on currently irrelevant information seems wiser than ignoring it altogether. This is because the real world outside of psychological laboratories often imposes dilemmas on human decision-making (20), which call for the balancing of antagonistic requirements. Complete persistence on one goal and one set of information might sometimes be beneficial, but on other occasions flexibility (i.e., the opposite of persistence) is needed. Relatedly, exploiting an ecological niche or one’s acquired knowledge might be a good strategy under some conditions, but exploring new niches and acquiring new knowledge might be smarter under others. Speed may sometimes be crucial, but sometimes accuracy is more important. Hence, the real-life challenges to cognitive control a more complex and more dynamic than a unipolar focus on persistence suggests.

This insight has had considerable impact on theorizing and research about cognitive control. According to the metacontrol model of Hommel (14) and Hommel and Colzato (15) [for similar approaches, see also (21–24)], people can vary in their cognitive processing style on a dimension characterized by its two poles: “persistence” on the one hand and “flexibility” on the other. Whereas a high degree of persistence corresponds to the original idea of cognitive control as willpower, with a strong focus on one goal and the information related to it, a high degree of flexibility is characterized by a more integrative, less selective and exclusive processing style, which facilitates switching between tasks, ideas, and actions, and taking into consideration a broader range of possibilities (14, 15, 20). In sum, the metacontrol model follows a bipolar logic: cognitive control arises from an effective, situationally adequate equilibrium between the opposing poles of the persistence-flexibility dimension. This equilibrium resembles a trade-off between these opposing poles in a way that a more stable cognitive persistence comes at the costs of cognitive flexibility and the opposite holds for an enhanced cognitive flexibility which comes at the price of cognitive persistence.

With respect to processing characteristics, persistence and flexibility are assumed to be characterized by the degree to which processing is under the control of the current goal/s and by the degree of competitiveness. As per Bogacz (25), optimal decision-making is driven by the currently active goals and is competitive in nature (winner-takes-all). This means that as one alternative is actively activated, other alternatives will tend to be less preferred or activated. For example, if Alexis hesitates between preparing an essay for school or streaming a TV show, the stronger activation of preparing an essay alternative will decrease the activation of the streaming preference. That is, if Alexis’s goal is to pass her English test, the essay alternative will be supported, and this will help her to inhibit the streaming preference. A central aspect of metacontrol model is that the degree to which alternatives compete and the amount of support offered by goals is determined by the present metacontrol state. Persistence is assumed to be characterized by a strong impact of the current goal/s and by a high degree of competitiveness, whereas flexibility is assumed to be characterized by a weak impact of goal/s and mild competitiveness. Getting back to our example, a strong persistence state would thus make Alexis hesitate between preparing the essay and streaming, and ultimately end up in a straight decision regarding one of the two options, possibly guided by the goal to pass the English test. On the other hand, a strong flexibility state might induce Alexis to try to combine the two options by reading the essay while streaming.

The metacontrol model is well supported by the empirical findings, as summarized and integrated elsewhere (2, 15). However, one observation is of particular importance for our purposes: the presence of inter-individual differences in metacontrol bias, and the impact of such differences on performance in various kinds of tasks. Inter-individual differences have been shown as a function of genetic predisposition and of cultural background. Genetic predisposition is assumed to affect metacontrol because of its apparent dependency on frontal and striatal dopaminergic pathways. Persistence seems to depend on frontal dopaminergic circuits, either because of the functional role of the frontal lobe that is fueled by the ventral tegmental area (VTA) *via* the frontal dopaminergic pathway (22) or because of the predominance of dopaminergic D1-family receptors in the frontal lobe (24). Flexibility, in turn, seems to depend on striatal dopaminergic circuits, either because of the functional role of the striatum fueled by the substantia nigra via the striatal dopaminergic pathway (22) or because of the predominance of dopaminergic D2-family receptors in that pathway (24, 26). Whether the systems are more aptly defined in terms of neuroanatomical site or receptor families, this suggests that there is a persistence-promoting system and a flexibility-promoting system that together (according to their relative strength or contribution) generate cognitive control as an emerging property, which can be biased toward persistence or toward flexibility. Accordingly, genetic predispositions that impair the optimal functionality of dopaminergic processing in the persistence system (frontal pathway or D1 receptors) should lead to poor performance in tasks that rely on persistence, whereas predispositions impairing the optimal functionality of dopaminergic processing in the flexibility system (striatal pathway or D2 receptors) should lead to poor performance in tasks relying on flexibility—which is indeed what the data show (15, 26–29). Culture can also play a role, as shown by findings that cultural factors that favor individualism are beneficial for performance in persistence-heavy tasks while factors that favor collectivism are beneficial for performance in flexibility-heavy tasks (15).

The interesting implication of these findings is that none of the investigated factors, whether biological or cultural in nature, was found to impair all kinds of performance of a particular sort. Rather, it was the nature of the task and its reliance on particular metacontrol states (persistence vs. flexibility) that determined whether a particular individual characteristic was beneficial or detrimental. In general, any bias toward persistence is beneficial for tasks that require a strong focus, that emphasize the distinction between task-relevant and task-irrelevant information and what one may call convergent thinking (30, 31), while any bias toward flexibility is beneficial for tasks that call for the integration of a broader range of information with different degrees of relevance, and for the broad use of information and internal concepts for test with high uncertainty—divergent thinking in terms of Guilford. Of particular interest for our purposes, almost all tasks that are clinically used to diagnose particular psychiatric conditions, such as the Stroop task, flanker tasks, or inhibition tasks, can be characterized as persistence-heavy/convergent, whereas hardly any task used in the clinical context of cognitive control emphasizes or relies on flexibility/divergent thinking. This raises the possibility that diagnosed individuals that are assumed to suffer from what is considered cognitive-control deficits may not, or not necessarily suffer from a general impairment of cognitive control, but rather from a selective difficulty to bias their metacontrol toward persistence. Flexibility-heavy tasks may in turn not show any control deficits and perhaps even demonstrate super-normal performance. Before we will discuss whether this speculation is supported by available findings, let us first apply the bipolar reasoning underlying metacontrol to psychiatric conditions.

## Toward Bipolar Clinical and Psychiatric Theorizing

**FIGURE 1:**
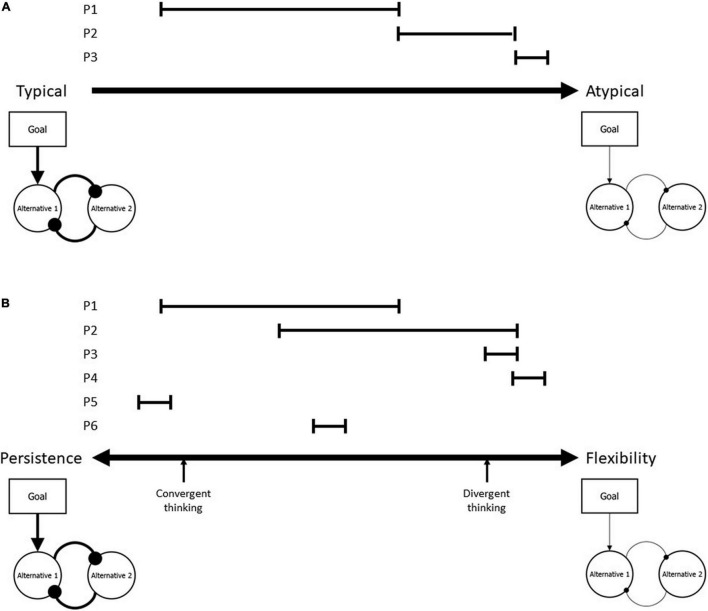
The implications of the classical unipolar view on psychiatric conditions **(A)** and the bipolar alternative developed in this article **(B)**. **(A)** Considers the average functioning of individuals as “typical” or “normal,” based on population means, and systematic deviation from these means as “atypical” or “deviant.” According to this logic, individual performance with a range exhibited by P1 would be likely to be diagnosed as typical, performance exhibited by P2 as borderline, and performance shown by P3 as sufficiently deviant to be psychiatrically relevant. The figures below the origin and the endpoint of the dimension characterize the processing style assessed by typical cognitive-control tasks, which are commonly biased toward persistence. Accordingly, persistent individuals have a higher probability to be considered typical than more flexible individuals. **(B)** Keeps these processing characteristics but turns the unipolar dimension into a truly bipolar, ranging from persistence to flexibility, with no evaluative meaning attached to either pole. P1–P6 characterize performance variability of individuals, as described in the text. Note that persistence is considered to be beneficial for some tasks, such as convergent thinking, while flexibility is considered to be beneficial for other tasks, such as divergent thinking.

Let us consider what a more bipolar account along the lines of theorizing in cognitive control would imply for the clinical sciences and let us begin with characterizing the established unipolar perspective on clinical conditions. This is sketched in Figure 1A, which locates three individuals (P1, P2, and P3) on a unipolar dimension ranging from “typical” to “atypical” (or normal to deviant). The behavior of individuals, whether mental processes or overt behavior, is dynamic and shows considerable intra-individual variability, which is why we characterize the behavior of individuals by ranges rather than single points on the dimension. P1 would be considered typical or normal. While the behavior of this individual would sometimes be very close to the population average and on other occasions be closer to the deviant area, the mean behavior of P1 would not be considered deviant or clinically problematic. An example of what the dimension refers to may be distractibility. We all have experienced situations where we are fully focused on the present task and find it easy to ignore distracting events, such as music playing in the background (which would locate our behavior close to the left pole) and other situations where we are easily distracted and find it hard to focus (which would locate us more to the right). However, many would never reach an area where they completely fail to focus on what is currently relevant to a degree that some ADHD patients, say, may exhibit. P3 may be exactly such a patient. Even this patient shows some variability, even this patient has good days and bad days. But the mean performance would indicate clearly deviant performance. Finally, P2 would show what often is called borderline performance: performance that often covers “non-deviant” area but sometimes approaches the “problematic” end of the dimension.

Given that we have characterized the tasks typically used to diagnose cognitive-control problems as persistence-heavy, as they emphasize the focusing on relevant issues and the active ignorance or suppression of irrelevant issues, we have characterized the “typical” origin of the dimension accordingly (see “bubbly” figure below the “Typical” origin of the dimension). This figure translates the decision-making core model discussed by Bogacz (25) into a particularly persistent version [cf. (15)]. A persistence bias of metacontrol would impact decision-making in two ways: by strengthening the impact of the present goal (which defines the relevance of information) on the decision-making process and by increasing the conflict between alternatives considered for a decision [i.e., by increasing the mutually inhibitory impact of one alternative on the other(s)]. This renders decision-making highly selective and exclusive. In contrast, the “Atypical” end of the dimension is characterized by flexibility. Accordingly, the figure shows a weak impact of the present goal and only weak competition between alternatives. This metacontrol state would lead to weak performance in a classical clinical cognitive-control task, like the Stroop task, but to strong performance in flexibility-heavy tasks, like brainstorming or information-integration tasks.

Once we have characterized the endpoints of our dimension in terms of their cognitive-functional implications, and once we have considered that being located close to one of the endpoints may disqualify an individual for some tasks but qualify her for others, we are ready to drop the normative, evaluative labeling of the dimension and make it truly bipolar. Panel B sketches the result. While the processing-related functional implications in the figures below the two endpoints remain the same, the left endpoint is now characterized as persistence and the right as flexibility, so that the dimension becomes a metacontrol dimension with two equally valuable poles. Even a perfectly “typical” individual might exhibit a performance range that is shifted to the left (P1) or right (P2) from the middle, as suggested by the research on interindividual differences in metacontrol (15). “Borderliners” may exist on both sides of the dimension, that is, close to the persistence pole or close to the flexibility pole, like P3. Even more “atypical” individuals, with rather little variability in their performance far from the mean, could be located on the bipolar dimension, but now it makes sense to consider far-from-the-mean performance for both sides of the dimension, like indicated for P4 and P5. And, of course, it is possible that even “typical” individuals might differ in terms of variability, as indicated for P6.

The interesting, novel implication of this bipolar approach is that theoretically and practically characterizing individuals on a bipolar persistence/flexibility dimension opens the possibility that weak performance on tasks that are biased toward one or the other pole may come with strengths on tasks that are biased toward the other pole. This perspective no longer requires us to consider some people less normal and less typical than others but provides a solid theoretical and mechanistically transparent basis for considering “atypical” individuals as “specially talented.” According to this metacontrol-based logic, the adaptivity of a particular processing style relies on the circumstances and on the task: while a strong bias toward persistence might be suboptimal for dealing with some tasks, it may be optimal for dealing with others (32).

So far, our aim was to argue that the difference between the two panels in Figure 1 consists in more than merely relabeling a dimension. Rather, we believe that the suggested bipolar approach opens new avenues for understanding and dealing with individual variability. However, before getting to these options, we first want to provide more evidence for the empirical basis and feasibility of our approach by applying it to two neurodevelopmental conditions: ASD and tic disorders. Let us thus take these two examples to see whether the idea that clinical atypicality and deficits in tasks related to one pole of a metacontrol dimension may come with strengths in tasks related to the other is realistic at all.

## Evidence From Autism Spectrum Disorder and Tic Disorders

Our approach suggests that people with a strong bias toward flexibility, which is likely to be present in many, but not all psychiatric conditions with a link to cognitive control, do not only show impairments in the persistence-heavy tasks that are commonly used to diagnose the condition, but might also excel in flexibility-heavy tasks that are commonly not considered in diagnosis. Elsewhere, we have discussed available evidence suggesting that this might indeed be true for people diagnosed as ADHD (32), but there is also evidence suggesting a flexibility bias in people with tic disorders. On the one hand, these individuals show worse performance than healthy controls in persistence-heavy tasks that emphasize and rely on the distinction between relevant and irrelevant information, as in tasks requiring the suppression of irrelevant stimuli (33), the suppression of irrelevant stimulus-response bindings (34), and the selective maintenance of memory information (35). In line with our hypothesis, however, people with tic disorders exhibit excellent, above-average performance in tasks that rely on flexibility, such as switching between tasks (36), strategies (37), and behaviors (38).

The opposite pattern (weak performance on flexibility-heavy tasks coming with strong performance on persistence tasks) has also been found, such as in patients with ASD. On the one hand, deficits in flexibility can be considered a defining feature of ASD, which is associated with deficits in social interaction and both verbal and non-verbal communication (39, 40) and in various cognitive tasks that rely on flexibility (41). For instance, two independent meta-analysis indicated that, across the life-span, people diagnosed with ASD perform less accurate and make more perseverative errors than healthy controls in the Wisconsin Card Sort Task, a task where the rules determining the correct response change over time without notice (42, 43). Along the same lines, a meta-analysis showed people with ASD to display difficulties in performing the Trail Making test, a task in which participants are required to switch from drawing lines between numbers and letters (44). Another meta-analysis indicated that people diagnosed with ASD generate fewer novel ideas than controls (45) and a systemic review revealed that people with ASD perform worse in task-switching paradigms [under time pressure; (41)]. On the other hand, however, ASD is characterized by stereotypic or repetitive behavior, and strict rituals, suggesting that ASD may be beneficial for performance on persistence-heavy tasks (41). Indeed, people diagnosed with ASD show enhanced performance in tasks that rely on persistence, such as resolving anagrams (46) and convergent thinking (45). Further, a systemic review revealed that people diagnosed with ASD outperform healthy control in perceptual discrimination (47). This enhanced perceptual discrimination in visual tasks has been interpretated as a superior ability to visually discriminate between targets and distractors (47), a typical process sustained by cognitive persistence.

In sum, research on tic disorders and ASD provides solid evidence in support of our claim that the performance deficits associated with neurodevelopmental atypicality on some tasks often come with strengths in other tasks. In particular, this research suggests that our characterization of the particular kind of atypicality in terms of persistence and flexibility biases, and the particular kind of diagnostic task, can capture the patterns exhibited by the available evidence.

## Conclusion

Traditional clinical and psychiatric accounts of mental health are based on a unipolar logic exclusively concentrating on cognitive impairments associated with clinical conditions. Unfortunately, this unipolar logic tends to stigmatize and discourage diagnosed individuals and ignores possible benefits coming with the diagnosed atypicality. Based on the metacontrol model, we call for a bipolar account of clinical sciences—an approach that focuses on possible cognitive gains, rather than insufficiencies, and that calls for more attention to the fit between the cognitive strengths of individuals and corresponding tasks and contexts fitting these strengths. This is not to say that for each individual performance profile there is necessarily a wide range of performatory options, especially if it is small in scope and lies close to a pole. While a high degree of persistence can be of great use for many tasks and activities, extreme forms (rigidity in clinical terms) may still be useful, but probably for rather special circumstances (e.g., for endurance sports or particularly demanding research expeditions) and opportunities that are so rare that they won’t be of much use for the respective individuals. The same holds for flexibility, which in particularly extreme forms might generate very innovative ideas that nevertheless fail because of insufficient persistence to turn them into reality. Nevertheless, we suggest that trying to identify and develop all available talents is an interesting clinical and societal alternative than to merely try reducing diagnosed atypicalities. Accordingly, this novel framework will be likely to encourage and fortify the self-esteem in diagnosed individuals and will potentially increase the positive view of neurocognitive atypicality.

## Data Availability Statement

The original contributions presented in this study are included in the article/supplementary material, further inquiries can be directed to the corresponding author/s.

## Author Contributions

All authors listed have made a substantial, direct, and intellectual contribution to the work, and approved it for publication.

## Conflict of Interest

The authors declare that the research was conducted in the absence of any commercial or financial relationships that could be construed as a potential conflict of interest.

## Publisher’s Note

All claims expressed in this article are solely those of the authors and do not necessarily represent those of their affiliated organizations, or those of the publisher, the editors and the reviewers. Any product that may be evaluated in this article, or claim that may be made by its manufacturer, is not guaranteed or endorsed by the publisher.
